# Heritability and evolvability of morphological traits of Savannah sparrows (*Passerculus sandwichensis*) breeding in agricultural grasslands

**DOI:** 10.1371/journal.pone.0210472

**Published:** 2019-01-14

**Authors:** Jenna A. Cava, Noah G. Perlut, Steven E. Travis

**Affiliations:** 1 Department of Biology, University of New England, Biddeford, Maine, United States of America; 2 Department of Environmental Studies, University of New England, Biddeford, Maine, United States of America; National Cheng Kung University, TAIWAN

## Abstract

Heritability and evolvability estimates of adult traits from free-living bird populations can be used to gauge the ability of populations to respond to selection, but are rare due to difficulties in gathering detailed pedigree information. The capacity to respond to selection is particularly important for species occupying managed habitats such as agricultural grasslands because of the potential for humans to accidentally influence traits. We calculated heritability and evolvability of six morphological traits in a population of Savannah Sparrows (*Passerculus sandwichensis*) breeding in a large agricultural landscape. We used microsatellite analysis to determine a genetic pedigree, revealing a high level of extra-pair paternity (63%) within a relatively philopatric population. For the entire population, heritabilities varied from low to high (bill width: 0.160±0.182 to tarsus length: 0.651±0.155), while evolvabilities were low across all traits (wing length: 0.035±0.013 to body mass: 0.066±0.106). Our results indicate that any directional selection from agricultural management practices will produce negligible changes in basic morphometrics of Savannah sparrow populations occupying the Champlain Valley of Vermont, USA.

## Introduction

Human habitat alteration and other management activities are known to affect several evolutionary processes in birds, such as restricting or enhancing gene flow between populations [1], and altering the selective pressures acting on individuals through food limitation (e.g., [2]) or novel predator introduction [3]. Less is known about how humans directly influence the evolution of populations through accidental selection on traits, despite the fact that most populations are probably under such selective pressures given current estimates that > 75% of ice-free land has been altered by human activity [4]. Understanding the potential evolutionary response of traits to relatively novel human selective pressures would provide information on how humans could influence the evolutionary trajectories of populations within altered landscapes.

Two quantitative genetic parameters have previously been used to measure evolutionary potential at the population level: heritability and evolvability [5]; [6]. Narrow-sense heritability (*h*^2^) is the proportion of a trait’s total phenotypic variance, *V*_*P*_, that is attributable to additive genetic variance, *V*_*A*_ [5]. While heritability is helpful for understanding the relative influences of genetics and the environment on the variation of a trait and is the more traditional measure of evolutionary potential, it is not useful for comparisons between traits because of large trait-by-trait differences in the amount of residual, or unexplained, variance [6]. Instead, evolvability (*I*_*A*_), defined as the percent change in a trait per unit change in the strength of selection and calculated by dividing *V*_*A*_ by the mean square of the phenotype, is more appropriate for comparisons across traits. Not only does it avoid the inconsistencies inherent in heritability estimation, it is also easier to interpret from an evolutionary perspective [6]; [7]. Both estimators have been used as measures of how populations of various taxa respond to different selective pressures varying across environments, and have provided the basis for several meta-analyses of micro-evolutionary potential [6]; [8]. However, few studies have applied both of these estimators to free-living bird populations because of difficulties collecting the phenotypic and pedigree information necessary for their calculation, particularly considering genealogy tracking is nearly impossible for natal dispersers of noncolonial, migratory species that rarely return to study areas (but see [9] for a broad review of heritabilities only).

The ‘animal model’ can estimate heritability and evolvability parameters for unbalanced and/or incomplete pedigree datasets collected from wild populations [10]. This modeling approach generates variance components for individual traits using restricted maximum likelihood methods. However, the application of this method still requires tracking individuals from birth to adulthood, so that both their parentage and trait values as adults can be quantified. Such datasets for highly mobile, migratory avian species are rare due to frequent difficulties tracking individuals from the nestling stage through adulthood, especially if natal dispersal rather than philopatry is the norm [11]. Many estimates of quantitative genetic parameters come from long-term (>15 years) studies of species that are colonial (e.g., [12]), are constrained to a very narrow habitat (e.g. [13]; [14]; [15]), or populations that are isolated on islands (e.g., [16]). Furthermore, none of the populations studied thus far have mostly resided within managed habitats, even though the vast majority of terrestrial habitats are managed [4].

Grassland birds, in particular, are under selective pressure from human activity because most of their native habitat has been replaced with row crops or intensely managed hayland and pasture [17]. In these grass-based systems, the timing and intensity of management are strong selective forces because they largely determine individual reproductive success, influence apparent survival and recruitment rates, and alter social and genetic mating systems [18]; [19]; [20]. Considering that migratory bird evolution in these landscapes is likely being shaped by human-mediated selection, future conservation efforts may depend on our ability to understand how traits may respond. Our objective was to generate the first heritability and evolvability estimates for a set of commonly measured morphological traits in a migratory passerine, the Savannah Sparrow (*Passerculus sandwichensis*), breeding in an agriculturally managed grassland landscape. These estimates will provide an important early indication of the potential for quantitative traits to evolve under continued selection from agricultural management, and will lay the groundwork for future studies of traits with more intimate links to individual fitness.

## Materials and methods

### Study site and field sampling

Appropriate animal care was approved under the University of New England IACUC protocol number UNE010-2009, and banding under U.S. federal permit 17610#23540. Our research took place during 2002–2014 within the Champlain Valley of Vermont, USA, a relatively isolated region containing approximately 146,000 ha of managed grassland [21]. In this region, up to 70% of available grassland habitat is managed during the breeding season [22]. We established two study areas on private lands, one at Shelburne (2002–2014, 44° 23' 40.542", -73° 15' 30.7908") and one at Hinesburg (2002–2009, 44° 20' 37.18", -73° 09' 57.65"), both of which consisted of a mosaic of grasslands, forest, and human developed areas. The owners of the lands gave permission to conduct the study on their respective sites. All grasslands in and around the study sites were divided into agricultural fields and managed under schemes known to strongly affect reproductive success, survival, and recruitment of our study population [18]; [19]; [23] for further demographic details and for information on natal dispersal and maps of the study region see [24]; [25].

We collected morphometric and genetic data on the Savannah Sparrows at five and four focal study fields at Shelburne and Hinesburg, respectively. The study fields were under diverse management schemes, which varied from low to high intensity depending on how often hay was harvested, where high intensity was characterized by both earlier and more frequent harvests or by being grazed by cows [18]. Two of the nine fields were under low-intensity management, which allowed for relatively high average reproductive success; two fields were under moderate-intensity management, which allowed for moderate or average reproductive success; three fields were under high-intensity management, which caused low-to-average reproductive success; and two fields were switched from high to low intensity management during the study [18]; (see [23] for further details). Samples were pooled across management types to estimate evolutionary potential for the entire Champlain Valley population.

Each year during early- to mid-May, we passively netted adult birds with an array of 20–24 mist nets 1–3 times at each field. We banded all adults with a unique combination of three color bands and one metal US Geological Survey (USGS) band, and measured six morphological traits: overall body mass, wing length, tarsus length, and bill length, depth, and width. One observer (N. Perlut) took >99% of all measurements. We also collected a small blood sample (20–60 μL) from the brachial vein. Blood was dried onto Whatman disc filter paper and stored in a bag with silica desiccation beads.

To help inform our pedigree analysis, we attempted to locate and record GPS coordinates of all nests on all study fields beginning in mid-May and ending in early-August. We determined female association by flushing them from the nest, and male association by observation of nestling provisioning and territory defense by males. If an adult associated with a nest remained unbanded, we attempted to capture it at the nest using mist nets. We checked nests every 1–2 day(s). When the nestlings reached 6–7 days of age we banded them with a metal USGS band and collected blood samples.

To increase our sample of birds with known parents, we searched for natal dispersers across years using binoculars, identifiable by their single metal band. We searched weekly on focal Shelburne fields in all years and focal Hinesburg fields during 2002–2009. We also searched all fields within a 1.5-km radius of Shelburne focal fields during 2005 to 2014, and all fields within a 1.5-km radius of Hinesburg focal fields in 2006; see [24] for details. In 2014 we expanded our search to fields that were ≥ 10 acres within a 10 km radius of the Shelburne focal fields (n = 88 fields, 1110.11 ha). When a natal disperser was identified, we recaptured it, gave it a unique color band combination, measured morphometrics (as described above), and collected a blood sample.

### Pedigree determination

We collected adult morphometrics on 95 locally hatched individuals. Egg dumping is not known in this species, so we were generally able to determine the identity of genetic mothers through field observations of incubation and feeding [20]. However, extra-pair paternity occurs at a high rate in our study population, with over half of offspring sired by extra-pair males (54%, [20]), thus requiring genetic analysis for accurately determining paternity (see [25] for exclusion probabilities). We confirmed paternity whenever possible through microsatellite analysis (see below), although we were not able to determine paternity for all individuals due to limited resources. We confirmed both parents for 42 individuals, only maternity for 46 individuals, and only paternity for 1 individual. These data allowed us to develop a pedigree covering three generations based on 187 individuals (37% of the study population), aided by the fact that our study population has demonstrated a relatively high rate of natal philopatry [24]; [25]. We note that data on some traits are missing for a small number of the individuals, so sample sizes are somewhat less than 187 for some traits (See S1 Table for PEDANTIC pedigree summary).

We performed paternity analysis using seven hypervariable microsatellite loci: *Psa*12 and *Psa*29 developed from *P*. *sandwichensis* [26]; *Escu*6 from *Emberiza schoeniclus* [27]; *Mme*1 and *Mme*8 from *Melospiza melodia* [28]; and *Psap*61 and *Psap*335 from *P*. *s*. *princeps* [29]. All PCR reactions were run at a total volume of 15 μL and contained 1 μL of 50 ng/μL DNA, 0.5 U Taq DNA polymerase, and 1X PCR Buffer (Invitrogen, Inc., Carlsbad, CA). Locus-specific annealing temperatures and concentrations of MgCl_2_, dNTPs, Primers, and BSA are provided in Table 1. Microsatellite alleles were sized and scored using GENEMAPPER, version 3.7 (Applied Biosystems Inc., Foster City, CA).

**Table 1:** Microsatellite locus-specific annealing temperatures and concentrations of MgCl_2_, dNTPs, Primers, and BSA for PCR reactions used in determining paternity of Savannah Sparrows.

Locus	Annealing Temp (C)	MgCl_2_ (mM)	dNTPs (mM)	Primers (each, μM)	BSA (μg/μL)
*Psa*12	60	8.3	0.3	1.06	-
*Psa*29	62	4.16	0.1	0.83	0.1
*Psap*61	62	4.0	0.2	0.2	0.1
*Psap*335	63	4.0	0.2	0.2	0.1
*Escu*6	59	5.7	0.3	1.06	-
*Mme*1	57	5.0	0.3	1.06	0.1
*Mme*8	65	4.0	0.3	1.06	-

We used CERVUS software to estimate null allele frequencies, to test for Hardy-Weinberg equilibrium allele frequencies, and to calculate exclusion probabilities (Table 2). The estimated null allele frequencies of two of the loci analyzed (*Mme*1 and *Psap*61) were higher than 0.05, so they were not used in the paternity analysis (Table 2). However, the remaining set of five loci were sufficient for determining paternity, with a combined exclusion probability of *p* = 4.2x10^-4^. We used CERVUS to assign paternity at the 95% confidence level using the four autosomally-inherited loci *Psa*12, *Escu*6, *Mme*8, and *Psap*335 [30]. When CERVUS identified two equally likely fathers that matched an offspring at all loci, we additionally used the Z-linked *Psa*29 to determine the identity of the father.

**Table 2:** Summary microsatellite data from paternity analysis of Savannah Sparrows (*H*_*e*_ = expected heterozygosity, *H*_*o*_ = observed heterozygosity, *p* = probability of false exclusion).

Locus	No. Alleles observed	*H*_*e*_	*H*_*o*_	*p*	Null allele frequency	H-W Test *p*-value
*Psa*12	10	0.76	0.75	0.452	0.002	0.89
*Psa*29^1^	32	0.95	0.92	0.111	0.015	0.92
*Psap*61	19	0.92	0.68	0.160	0.15	<0.01
*Psap*335	15	0.87	0.88	0.265	0	0.24
*Escu*6	18	0.91	0.88	0.179	0.014	0.19
*Mme*1	30	0.95	0.74	0.111	0.12	<0.01
*Mme*8	27	0.91	0.88	0.176	0.021	0.10

^1^Z-linked, calculated *H*_*e*_ and *H*_*o*_ for males only.

### Variance components, heritability, and evolvability estimation

We estimated variance components for each trait using restricted maximum-likelihood (REML) by fitting mixed-effect ‘animal models’ in the program WOMBAT [31]; [32]. We analyzed each trait separately. The same model was applied to all traits except for the model applied to mass, which included an additional fixed effect. This general model included sex as a fixed effect because males tend to be 5% larger than females [33], and year and ancestry as random effects. The model applied to mass also included the date of capture as a fixed effect because mass declined with date over the course of the breeding season in our population (β = -0.016, *r²* = 0.04268, *p* = 0.0004). We lacked sufficiently detailed pedigree information to estimate maternal or common environmental effects. While it is probable that maternal effects influenced morphometrics in our population considering that such effects were documented previously in an island population of Savannah Sparrows [16], it is unlikely they had a large impact on our heritability or evolvability estimates because a minority of the individuals (18%) in our dataset shared a mother (i.e., were full or maternal half-siblings with at least one other bird). We were also unable to age breeding adults hatched outside of the study area, so we could not include hatch year as a common environmental effect. Narrow-sense heritability estimates (*h*^2^) and their standard errors were estimated in WOMBAT. We calculated evolvability (*I*_*A*_) by dividing additive genetic variance (*V*_*A*_) by the mean square of the phenotype and multiplying by 100 [6]; [7]. Following [34], we used the R package PEDANTICS to assess statistical power.

## Results

### Paternity analysis

We were able to successfully determine the genetic father of43 of the 95 recruits considered in our animal model, although one of these recruits was dropped from further analysis because maternity could not be confirmed. Twenty seven (63%) of these 43 individuals were extra-pair young based on exclusion of the territorial male as the genetic father. In 4 additional recruits the presence of extra-pair young was suggested by exclusion of the territorial male as the genetic father, but no genetic father was identifiable on the study field. All mothers identified through field observations were confirmed by maternity analysis, although in the case of 1 recruit no mother was identifiable by either observational or genetic methods.

### Variance components, heritability, and evolvability

**Fig 1:**
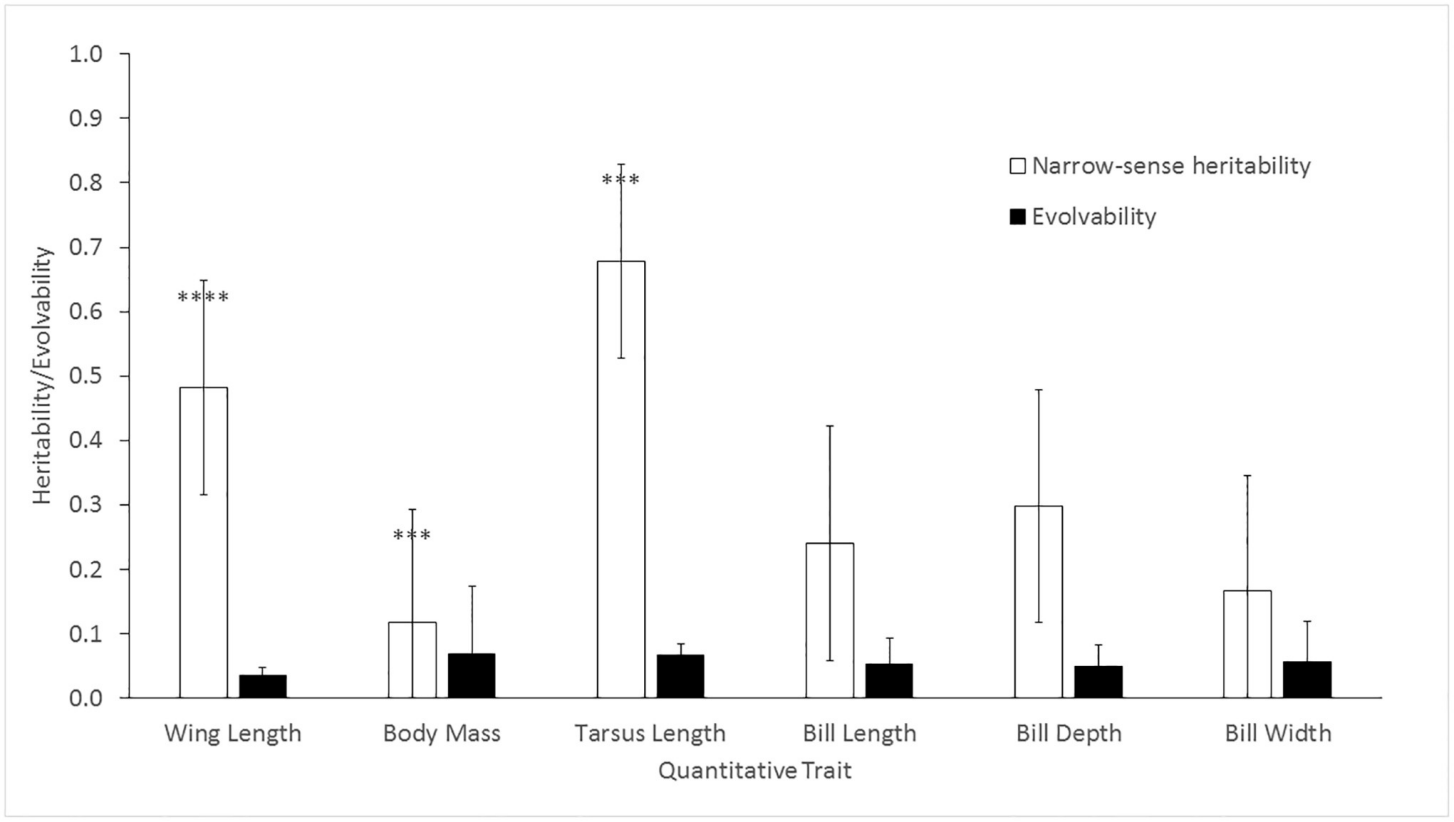
Heritabilities (*h*^2^) and evolvabilities (*I*_*A*_) of six morphological traits in Savannah Sparrows breeding in the Champlain Valley of Vermont, 2002–2014. Error bars are standard errors. Heritability estimates were tested for significance using likelihood ratio tests in WOMBAT (*p<0.05; **p<0.01; ***p<0.001; ****p<0.0001).

All variance components, phenotypic means, and sample sizes for the whole population are reported in Table 3 (note that sample sizes include both parents and their adult offspring, i.e., recruits, where parentage could be confirmed). Heritability varied widely among the six traits studied, with bill width having the lowest (*h*^*2*^ ± standard error: 0.160±0.182) and tarsus length the highest heritability (0.651±0.155; Fig 1). Tarsus and wing length had high heritabilities, with additive genetic effects accounting for over 50% of their variation, while body mass and the three bill measurements had relatively low heritabilities, ranging from 0.160–0.279 (Fig 1). Evolvability was low (less than 0.1%) for all traits, with wing length the lowest (0.035±0.013) and body mass the highest (0.066±0.106; Fig 1).

**Table 3:** Variance components, phenotypic means (and sample sizes (n) used in evaluating six morphological traits in Savannah Sparrows from the Champlain Valley of Vermont, 2002–2014 (*V*_*P*_ = Phenotypic variance =, *V*_*A*_ = additive genetic variance =, and *V*_*R*_ = residual variance).

Trait	*V*_*P*_	SE *V*_*P*_	*V*_*A*_	SE *V*_*A*_	*V*_*R*_	SE *V*_*R*_		SD	*n*
Wing	2.980	0.328	1.528	0.565	1.477	0.472	65.9 mm	2.451	182
Mass	1.931	0.206	0.228	0.341	1.703	0.373	18.2 g	1.442	180
Tarsus	0.406	0.047	0.275	0.079	0.130	0.059	20.4 mm	0.663	173
Bill Length	0.138	0.016	0.033	0.026	0.096	0.026	8.0 mm	0.388	175
Bill Depth	0.047	0.005	0.014	0.009	0.029	0.008	5.3 mm	0.222	173
Bill Width	0.064	0.007	0.011	0.012	0.050	0.012	4.3 mm	0.254	174

## Discussion

We estimated the evolutionary potential of morphological traits in a migratory population of Savannah Sparrows under selective pressure from agricultural management. Genetics had the greatest relative influence on the phenotypic variation of two indicators of overall body size, tarsus length and wing length, while it had less influence on bill dimensions. While heritability varied considerably among the traits studied, evolvability was relatively low for all of the traits.

The rate of extra-pair paternity among the relatively philopatric individuals detected in our study was high (63%), as expected from similarly high rates previously observed in this population (54% [20]; [25]). Other studies of migratory species have had to incorporate social data into their pedigrees to generate sufficiently large sample sizes; those cases assumed rare or no extra-pair paternity within their study populations in order to generate estimates [14]; [16]; we were able to account for the high rate of extra-pair paternity in our study population by way of genotyping using molecular markers, and thus were able to create an error-free pedigree for the individuals included. Use of pedigrees containing only social parent information is probably not a viable option for many studies on traits measurable only in adult passerines because even limited extra-pair paternity would have a strong effect on heritability estimates [14]. Furthermore, many migratory songbird populations show low return rates of natal dispersing birds, and thus require expanded search radii and/or many years of search efforts [11].

Heritability estimates of adult morphological traits are available for some songbird species; for example, they have been reported for the Medium Ground Finch (*Geospiza fortis*, [35]), Great Reed Warbler (*Acrocephalus arundinaceus*, [36]), House Sparrow (*Passer domesticus*, [37]), and an island population of Savannah Sparrows [16]. As expected when comparing across species whose traits may have different amounts of residual variance [6], there are no consistent patterns among the heritabilities of mass, tarsus length, bill length and bill depth, which are the traits most commonly studied [16]; [35]; [36]; [37]. Studies on heritability and evolvability of nestling traits (e.g., [14]; [38]) or adult fitness traits such as laying date, age of recruitment, and lifetime reproductive success (e.g., [39]) have also been conducted.

Teplitsky et al. [40] reviewed evolvabilities of four of the morphological traits measured in our study (mass, wing length, tarsus length, and bill length) for 10 wild bird populations, including Red-billed Gulls (*Chroicocephalus scopulinus*), Barn Swallows (*Hirundo rustica*), Collared Flycatchers (*Ficedula albicollis*), Great Reed Warblers, House Sparrows, and Blue Tits (*Cyanistes caeruleus*). Similar to our estimates, these morphological traits tended to have low evolvabilities (less than 0.1), although mass in some species was slightly more evolvable (0.1–0.2). The evolvability of several of the morphological traits measured in our study were also estimated by [16] for an isolated, island population of Savannah Sparrows living free from the influence of agricultural management. While [16] separated their estimates by sex and age class to investigate selection on each demographic whereas we investigated selection on our whole population, all of our evolvability estimates (except for bill width, which was measured only in this study) were similarly low (less than 0.1). The exceptions in [16] were body mass in males > 1-year old, in 1-year-old females, and in females > 1-year-old.

The similarities between published estimates of evolvability in birds suggest that most morphological traits have low capacities to respond to selection. Body mass seems to be a minor exception, with slightly higher evolvability in most of the species and populations studied. To put this result in a generational context, a change of 10% in mean mass would be expected to take 240 generations in populations where evolvability is relatively low (*I*_*A*_ = 0.04; [6]; [40]), whereas the same change would be expected to take 54 generations where evolvability is similar to our estimate (*I*_*A*_ = 0.178). Human land management, however, may disrupt (e.g., [20]) the evolutionary potential of such traits in populations using managed habitat. For example, management practices in our study area changed in both timing and intensity over the span of our long-term sampling, perhaps all but eliminating the potential for sustained directional responses of body mass to selection on those fields. Further studies on other species living on intensely managed areas are needed to determine whether such disruptions are typically the case.

In general, agricultural management has the potential to directly exert selection pressures on morphological traits. However, the most pronounced effects on fitness are likely to come from hay harvest or grazing events, which may be mostly nondiscriminatory with regard to morphological phenotypes, and which may actually dilute the effects of natural selective pressures. Nevertheless, one can envision specific scenarios under which birds with certain features, such as greater overall body mass, might gain advantages under agricultural management, such as the ability to initiate post-harvest nesting or to produce offspring that reach full size and develop flight more quickly. Unfortunately, our findings of low evolvability of morphological traits including body mass suggest a minimal potential to respond to management pressures in the near term, even if those pressures are consistent over evolutionarily meaningful time-spans. On the other hand, life-history traits such as the minimum time between reproductive events may have a much greater potential to respond to selection, considering that a short post-harvest period would necessarily select for birds capable of reproducing quickly following harvest events because only offspring that are produced during these constrained time-periods are able to recruit into this breeding population. Thus, future research should focus on the heritability and evolvability of other types of traits such as life-history and/or life-cycle traits to determine if and how they may respond to human activities, as well as the strength and direction of selection on both morphological and other traits, which were outside the scope of this study.

## Supporting information

S1 Table PEDANTIC pedigree summary for Savannah Sparrows breeding in the Champlain Valley of Vermont, 2002–2014. (DOCX)

S1 Data Data set used for this study. (XLSX)

## References

[1] Crispo E, Moore J, Lee-Yaw JA, Gray SM, Haller BC. Broken barriers: Human-induced changes to gene flow and introgression in animals. Bioessays 2011;33: 508–518. 21523794 10.1002/bies.201000154

[2] Ibáñez-Álamo JD, Soler M. Does urbanization affect selective pressures and life-history strategies in the common blackbird (*Turdus merula* L.)? Biol J Linn Soc 2010;101: 759–766.

[3] Urlich SC. What’s the end-game for biodiversity: is it time for conservation evolution? New Zeal J Ecol 2015;39: 133–142.

[4] Ellis EC, Ramankutty N. Putting people in the map: Anthropogenic biomes of the world. Front Ecol Environ 2008;6: 439–447.

[5] Falconer DS, Mackay TFC. Introduction to quantitative genetics, 4th edn Longman Group Ltd, Harlow; 1996.

[6] Hansen TF, Pélabon C, Houle D. Heritability is not evolvability. Evol Biol 2011;38: 258–277.

[7] Houle D. Comparing evolvability and variability of quantitative traits. Genetics 1992;130: 195–204. 1732160 10.1093/genetics/130.1.195 PMC1204793

[8] Garcia-Gonzalez F, Simmons LW, Tomkins JL, Kotiaho JS, Evans JP. Comparing evolvabilities: Common errors surrounding the calculation and use of coefficients of additive genetic variation. Evolution 2012;66: 2341–2349. 10.1111/j.1558-5646.2011.01565.x 22834736

[9] Postma E. Four decades of estimating heritabilities in wild vertebrate populations: improved methods, more data, better estimates? In: Charmantier Garant AD, Kruuk LEB, editors. Quantitative Genetics in the Wild. Oxford: Oxford University Press; 2014 Pp. 16–33.

[10] Kruuk LEB. Estimating genetic parameters in natural populations using the ‘animal model’. Philos T R Soc B 2004;359: 873–890. 10.1098/rstb.2003.1437 PMC1693385 15306404

[11] Weatherhead PJ, Forbes MRL. Natal philopatry in passerine birds: genetic or ecological influences? Behav Ecol 1994;5: 426–433.

[12] Teplitsky C, Mills JA, Yarrall JW, Merila J. Heritability of fitness components in a wild bird population. Evolution 2009;63: 716–726. 10.1111/j.1558-5646.2008.00581.x 19054048

[13] Kruuk LEB, Merilä J, Sheldon BC. Phenotypic selection on a heritable trait revisited. Am Nat 2001;158: 557–571. 10.1086/323585 18707351

[14] Charmantier A, Réale D. How do misassigned paternities affect the estimation of heritability in the wild? Mol Ecol 2005;14: 2839–2850. 10.1111/j.1365-294X.2005.02619.x 16029482

[15] Saunders SP, Cuthbert FJ. Genetic and environmental influence on fitness-related traits in an endangered shorebird population. Biol Conserv 2014;177: 26–34.

[16] Wheelwright NT, Keller LF, Postma E. The effect of trait type and strength of selection on heritability and evolvability in an island bird population. Evolution 2014; 68: 3325–3336. 10.1111/evo.12499 25130048

[17] Hannah L, Carr JL, Lankerani A. Human disturbance and natural habitat: a biome level analysis of a global dataset. Biodivers Conserv 1995;4: 128–155.

[18] Perlut NG, Strong AM, Donovan TM. Buckley NJ. Grassland songbirds in a dynamic management landscape: behavioral responses and management strategies. Ecol App 2006;16: 2235–2247. 17205901 10.1890/1051-0761(2006)016[2235:gsiadm]2.0.co;2

[19] Perlut NG, Strong AM, Donovan TM, Buckley NJ. Grassland songbird survival and recruitment in agricultural landscapes: implications for source-sink demography. Ecology 2008;89: 1941–1952. 18705380 10.1890/07-0900.1

[20] Perlut NG, Freeman-Gallant CR, Strong AM, Donovan TM, Kilpatrick CW, Zalik NJ. Agricultural management affects evolutionary processes in a migratory songbird. Mol Ecol 2008;17: 1248–1255. 10.1111/j.1365-294X.2008.03695.x 18302687

[21] NASS (National Agriculture Statistics Survey). 2010 [Cited 28 August 2013]. In: Census of Agriculture [Internet]. http://www.agcensus.usda.gov/index.php.

[22] Perlut NG, Strong AM, Donovan TM, Buckley NJ Regional population viability of grassland songbirds: effects of agricultural management. Biol Conserv 2008;141: 3139–3151.

[23] Perlut NG, Strong AM, Alexander TJ. A model for integrating wildlife science and agri-environmental policy in the conservation of declining species. J Wildlife Manage 2011;75: 1657–1663.

[24] Fajardo N, Strong AM, Perlut NG, Buckley NJ. Natal and breeding dispersal of Bobolinks (*Dolichonyx oryzivorus*) and Savannah Sparrows (*Passerculus sandwichensis*) in an agricultural landscape. Auk 2009;126: 310–318.

[25] Cava JA, Perlut NG, Travis SE. Why Come Back Home? Investigating the intrinsic and extrinsic proximate factors that influence natal philopatry in migratory passerines. Anim Behav 2016;118: 39–46.

[26] Freeman-Gallant CR, Wheelwright NT, Meicklejohn KE, States SL, Sollecito SV. Little effect of extra-pair paternity on the opportunity for sexual selection in Savannah sparrows (*Passerculus sandwichensis*). Evolution 2005;59: 432–440. 15807426

[27] Hanotte O, Zanon C, Pugh A. Isolation and characterization of microsatellite loci in a passerine bird–the reed bunting *Emberiza schoeniclus*. Mol Ecol 1994;3: 529–530. 7952335 10.1111/j.1365-294x.1994.tb00133.x

[28] Jeffery KL, Keller LF, Arcese P, Bruford MW. The development of microsatellite loci in the song sparrow, *Melospiza melodia* (Aves) and genotyping errors associated with good quality DNA. Mol Ecol 2001;1: 11–13.

[29] Temple M. Microsatellite analysis of extra-pair fertilizations in the Ipswich sparrow (Passerculus sandwichensis princeps). M.Sc. thesis, Dalhousie University. 2000.

[30] Kalinowski ST, Taper ML, Marshall TC. Revising how the computer program CERVUS accommodates genotyping error increases success in paternity assignment. Mol Ecol 2007;16: 1099–1106. 10.1111/j.1365-294X.2007.03089.x 17305863

[31] Meyer K. WOMBAT—digging deep for quantitative genetic analyses by restricted maximum likelihood. In: Proceedings of the 8th World Congress in Genetic Applied Livestock Production, Belo Horizonte, Brazil; 2006. pp. 27–14.

[32] Wilson AJ, Réale D, Clements MN, Morrissey MM, Postma E, Walling CA, et al An ecologist’s guide to the animal model. Journal of Anim Ecol 2010;79: 13–26. 10.1111/j.1365-2656.2009.01639.x 20409158

[33] Wheelwright NT, Rising JD. Savannah Sparrow (*Passerculus sandwichensis*) In: Poole A, Gill F, editors. The Birds of North America, No. 45. Washington, DC: Academy of Natural Sciences, Philadelphia, and American Ornithologists’ Union; 2008.

[34] Morrissey MB, Wilson AJ, Pemberton JM, Ferguson MM. A framework for power and sensitivity analyses for quantitative genetic studies of natural populations, and case studies in Soay sheet (*Ovis aries*). J Evol Biol 2007;20: 2309–2321. 10.1111/j.1420-9101.2007.01412.x 17956393

[35] Keller LF, Grant PR, Grant R, Petren K. Heritability of morphological traits in Darwin’s Finches: misidentified paternity and maternal effects. Heredity 2001;87: 325–336. 11737279 10.1046/j.1365-2540.2001.00900.x

[36] Åkesson M, Bensch S, Hasselquist D, Tarka M, Hansson B. Estimating heritabilities and genetic correlations: comparing the ‘animal model’ with parent-offspring regression using data from a natural population. PLoS ONE 2008;3(3): e1739 10.1371/journal.pone.0001739 18320057 PMC2254494

[37] Jensen H, Sæther BE, Ringsby TH, Tufto J, Griffith SC, Ellegren H. Sexual variation in heritability and genetic correlations in morphological traits in House Sparrow (*Passer domesticus*). J Evol Biol 2003;16: 1296–1307. 14640421 10.1046/j.1420-9101.2003.00614.x

[38] Kinnard TB, Westneat DF. Phenotypic and genetic variance of House Sparrows (*Passer domesticus*) early in development. Auk 2009;126: 884–895.

[39] Kim SY, Fargallo JA, Vergara P, Martínez-Padilla J. Multivariate heredity of melanin-based coloration, body mass and immunity. Heredity 2013;111: 139–146. 10.1038/hdy.2013.29 23591519 PMC3716269

[40] Teplitsky C, Tarka M, Moller AP, Nakagawa S, Balbontin J, Burke TA, et al Assessing multivariate constraints to evolution across ten long-term avian studies. PLOS ONE 2014;9: e90444 10.1371/journal.pone.0090444 24608111 PMC3946496

