# Supplementary material for: Heritability and evolvability of morphological traits of Savannah sparrows (Passerculus sandwichensis) breeding in agricultural grasslands
Source: PLoS One. 2019 Jan 14;14(1):e0210472. doi: 10.1371/journal.pone.0210472 (PMC6331091; doi:10.1371/journal.pone.0210472)
Supplement: S1 Table — (DOCX) [file pone.0210472.s001.docx]

**Supporting Information**

**S1 Table. PEDANTIC pedigree summary for Savannah Sparrows breeding in the Champlain Valley of Vermont, 2002-2014.**

| Records | 187 |
| --- | --- |
| Maternities | 88 |
| Paternities | 42 |
| Full Siblings | 5 |
| Maternal Siblings | 24 |
| Maternal Half Siblings | 19 |
| Paternal Siblings | 12 |
| Paternal Half Siblings | 7 |
| Maternal Grandmothers | 5 |
| Paternal Grandmothers | 5 |
| Paternal Grandfathers | 3 |
| Paternal Grandfathers | 3 |
| Max Pedigree Depth | 2 |
| Founders | 98 |
| mean maternal sibsip size | 1.294118 non-zero F 0.0 |
| mean paternal sibsip size | 1.3125 F > 0.125 0.0 |
| mean pairwise relatedness | 0.00461 |
| pairwise relatedness>=0.125 | 0.00461 |
| pairwise relatedness>=0.25 | 0.01029 |
| pairwise relatedness>=0.5 | 0.00776 |
